# Investigation of Zinc bis(1,4-didecylbenzo)-bis(2,3-pyrido) Porphyrazine for Application as Photosensitizer in Photodynamic Therapy of Cancer

**DOI:** 10.1155/2008/392090

**Published:** 2008-02-26

**Authors:** Keiichi Sakamoto, Eiko Ohno-Okumura, Taku Kato, Masaki Watanabe, Michael J. Cook

**Affiliations:** ^1^Department of Applied Molecular Chemistry, College of Industrial Technology, Nihon University, 1-2-1 Izumi-cho, Narashino-shi, Chiba-ken 275-8575, Japan; ^2^Research Institute of Chemical Science, Technology and Education, 8-37-1 Narashinodai, Funabashi-shi, Chiba-ken 274-0063, Japan; ^3^Nissan Chemical Industries, LTD. Electronic Materials Research Laboratories, 722-1 Tsuboi-cho, Funabashi-shi, Chiba-ken 274-8507, Japan; ^4^U-TEC Corporation, Innovation Technology Development, 21-1 Ohmori-cho, Nara-shi, Nara-ken 630-8131, Japan; ^5^School of Chemical Sciences and Pharmacy, University of East Anglia, Norwich NR4 7TJ, UK

## Abstract

The phthalocyanine analogue containing nonperipheral long alkyl-substituted benzenoid rings and pyridine rings, zinc bis(1,4-didecylbenzo)-bis(2,3-pyrido) porphyrazine, was synthesized. Zinc bis(1,4-didecylbenzo)-bis(2,3-pyrido) porphyrazine reacted with dimethyl sulfate and monochloroacetic acid to produce their quaternized products and diethyl sulfate to produce the sulfo-substituted products. All quaternized and sulfo-substituted showed amphiphilic character. Identical peaks in cyclic voltammograms appeared for these products before and after quaternization. During the evaluation of zinc bis(1,4-didecylbenzo)-bis(2,3-pyrido) porphyrazine for its photodynamic therapy of cancer (PDT) efficacy by cancer cell culture, the light exposed dimethyl sulfate quaternized zinc bis(1,4-didecylbenzo)-bis(2,3-pyrido) porphyrazines in IU-002 cells produce cell disruption that can be detected as a decrease in fluorescence.

## 1. INTRODUCTION

Phthalocyanine derivatives have attracted attention as functional chromophores for applications, especially organic charge carriers in photocopiers, as laser light absorbers in data storage systems, as photoconductors in photovoltaic cells, and in electrochromic displays [1–3]. Moreover, phthalocyanine derivatives can be utilized as sensitizers in photodynamic therapy of cancer (PDT).

Sensitizers for PDT require high photostability, high selectivity to tumors, no dark cytotoxity, strong absorption in the region between 600 and 800 nm where penetration of tissue is good, a long triplet lifetime, and satisfactory photosensitization of singlet oxygen. Phthalocyanine derivatives are known to satisfy the aforementioned conditions [3–8].

We previously synthesized the nonperipherally substituted phthalocyanine derivatives, zinc alkylbenzopyridoporphyrazines, which possessed didecylbenzenoid and pyridinoid moieties in the molecule and described regio isomer separation of one of the alkylbenzopyridoporphyrazines [9]. We reported a fundamental study on PDT by measuring for the triplet state lifetime of the alkylbenzopyridoporphyrazins and regio isomers [10, 11]. As alkylbenzopyridoporphyrazine exhibited solubility in organic solvents and was expected to have a higher tumor affinity, quaternation of the pyridine nitrogen in the alkylbenzopyridoporphyrazine was done to give solubility in an aqueous media, and to have bioavailability and in vivo distribution [12]. Then, Nyokong et al. reported that phthalocyanine analogues, tetra-2, 3-pyridoporphyrazine and its quaternized compounds have excellent properties compared to zinc phthalocyanine-type photosensitizer [13]. The amphiphilic phthalocyanine derivatives were concluded the best compound for a new generation of photosensitizers for PDT [12]. In our previous papers [9–12], the reported zinc bis(1,4-didecylbenzo)-bis(3,4-pyrido) porphyrazine and its regio isomers were prepared by 1 : 1 mixture of 3,6-didecylphthalonitrile and 3,4-pyridine dicarbonitrile.

In the present study, another type, novel nonperipheral, substituted phthalocyanine derivative, zinc bis(1,4-didecylbenzo)-bis(2,3-pyrido) porphyrazine was synthesized.

In the case of related compounds, 2,3-pyridoporphyrazines are known to have not only longer wavelength but stronger absorption intensity than corresponding phthalocyanines and 3,4-pyridoporphyrazines [14]. In accordance with [14], it is expected that zinc bis(1,4-didecylbenzo)-bis(2,3-pyrido) porphyrazine and its quaternation compounds have stronger absorption intensities than that of zinc bis(1,4-didecylbenzo)-bis(3,4-pyrido) porphyrazines reported before [9–12]. Therefore, the novel compound, zinc bis(1,4-didecylbenzo)-bis(2,3-pyrido) porphyrazine and its quaternation compounds are expected to be excellent photosensitizer for PDT.

## 2. RESULTS AND DISCUSSION

### 2.1. Synthesis and quaternization of phthalocyanine derivative

**Figure 1:**
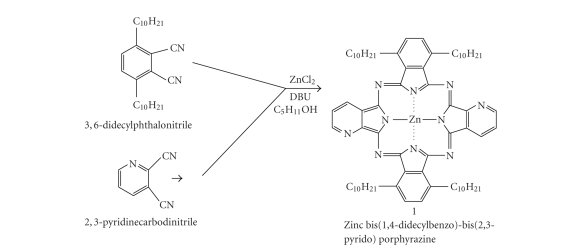
Synthetic pathway of zinc bis(1,4-didecylbenzo)-bis(2,3-pyrido) porphyrazine.

The synthetic procedure used to prepare the novel nonperipheral-substituted phthalocyanine derivative, zinc bis(1,4-didecylbenzo)-bis(2,3-pyrido) porphyrazine, was the same as that used for the preparation of zinc bis(1,4-didecylbenzo)-bis(3,4-pyrido) porphyrazine [9–12]. Zinc bis(1,4-didecylbenzo)-bis(2,3-pyrido) porphyrazine was synthesized in 80% yield using equimolar amounts of 3,6-didecylphthalodinitrile and 2,3-pyridine carbonitrile in the presence of 1,8-diazabicyclo[5.4.0]undec-7-ene (DBU) as basic catalyst (see Figure 1). The target compound, zinc bis(1,4-didecylbenzo)-bis(2,3-pyrido) porphyrazine, and its intermediates were studied using Fourier transformation infrared (IR), proton nuclear magnetic resonance (^1^H-NMR), ultraviolet-visible (UV-Vis) spectroscopy, and elemental analysis. The analytical data of the compound were in good agreement with the proposed structure.

The synthesized zinc bis(1,4-didecylbenzo)-bis(2,3-pyrido) porphyrazine was anticipated to be a mixture of products, with different numbers of pyridine rings in the molecule. However, the target compound comprised only the proposed constituent as confirmed by thin layer chromatography (TLC). As the target compound had been purified by TLC using benzene as eluent, only one blue-colored constituent was obtained. It is thought that the desired compound was obtained in accordance with the mole ratio of the raw materials used. The same phenomenon has been observed in the case of synthesis of zinc bis(1,4-didecylbenzo)-bis(3,4-pyrido) porphyrazine [9–12].

Zinc bis(1,4-didecylbenzo)-bis(2,3-pyrido) porphyrazine has two alkylbenzenoid and two pyridinoid rings in different locations; thus, it has five regio isomers, three of which have rings adjacent to the pyridinoido rings while the other two have opposed pyridinoid rings. Although we previously reported the separation of regio isomers in alkylbenzopyridoporphyrazine [9–12], no attempt was made in this work to isolate the isomers of zinc bis(1,4-didecylbenzo)-bis(2,3-pyrido) porphyrazine. Of course, the obtained blue-colored constituent will be further separated into five regio isomers by using toluene-pyridine 7 : 3 eluent in accordance with [9–12].

The zinc bis(1,4-didecylbenzo)-bis(2,3-pyrido) porphyrazine reacted with quaternizing agents such as monochloroacetic acid (MCAA), diethyl sulfate (DES), and dimethyl sulfate (DMS) in *N,N*-dimethylformamide (DMF) as a solvent at 140^°^C for 2 hours.

The respective products obtained were greenish-blue-colored powders of which the yields were 24, 21, and 25% for MCAA, DES, and DMS, respectively (see Figure 3). Zinc bis(1,4-didecylbenzo)-bis(2,3-pyrido) porphyrazine was dissolved in toluene, chloroform, pyridine, and methanol but not in water. After reacting with quaternizing agents, the products were also soluble in water.

In the cases of MCAA and DMS, analysis revealed that the structures of the products were in good agreement with those having *N*-CH_2_COOH and *N*-CH_3_ groups, respectively. Whereas when DES was used as a quaternizing agent, no *N*-CH_2_CH_3_ singlet peak was present in the ^1^H-NMR spectrum, S=O stretching in the IR spectrum was observed. Therefore, sulfonation but not quaternization was achieved [12, 15].

After reaction with the quaternizing agents, all compounds possessed amphiphilic properties.

### 2.2. Spectroscopic and electrochemical properties

The UV-Vis spectrum of zinc bis(1,4-didecylbenzo)-bis(2,3-pyrido) porphyrazines around 700 nm is characteristic of phthalocyanine analogues, with the Q band attributable to the difference between the highest occupied molecular orbital (HOMO) energy level and the lowest unoccupied molecular orbital (LUMO) energy, that is, the *π*-π* transition of the phthalocyanine ring.

The quarternized derivatives of zinc bis(1,4-didecylbenzo)-bis(2,3-pyrido) porphyrazines showed strongest absorption at 676, 687, and 687 nm in water after reaction with DMS, DES, and MCAA, respectively (Table 1); these Q bands were bathochromic compared to the nonquaternized parent compound. As the UV-Vis spectra of the quaternized compounds in water showed very broad peaks, the amphiphilic compounds had excellent molecular association tendency.

Zinc bis(1,4-didecylbenzo)-bis(2,3-pyrido) porphyrazine and its quaternized compounds fluoresced on exposure to ultraviolet light. Although fluorescence spectra generally were known to be mirror images of UV-Vis spectra at the longer wavelengths, the Q bands nearly overlapped with the wavelengths at which fluorescence occurs in the case of zinc bis(1,4-didecylbenzo)-bis(2,3-pyrido) porphyrazine and its quaternized compounds, thus, the differences between *λ*_max_ of UV-Vis and the *F*_max_ of fluorescence spectra, called the Storkes shift, were very small between 10 to 20 nm. These observations are similar to that seen with the phthalocyanines zinc bis(1,4-didecylbenzo)-bis(2,3-pyrido) porphyrazine and its quaternized derivatives. These compounds are molecules with high planarity which cannot change their configuration after quaternization.

The important parameters of a cyclic voltammetry (CV) are the reduction (*E*_*pc*_) and oxidation (*E*_*pa*_) potential, the difference between reduction and oxidation potentials, (Δ*E*_*pc*_), and formal reduction potential (*^E°'^*) of the observed waves. [Note: *E*_mid_=*^E°'^*] (Table 2 and see Figure 3).

The potential difference in CVs between the reduction and oxidation correspond to the HOMO-LUMO energy gaps of the compound [16]. Just as chemical reactions occur during the electron transfer between HOMO and LUMO energy levels, photochemical reactions are also based on similar phenomena of energy transfer. Before and after the quaternization, the HOMO-LUMO energy gap of zinc bis(1,4-didecylbenzo)-bis(2,3-pyrido) porphyrazine was unchanged. The shapes of CVs clearly showed that quaternized zinc bis(1,4-didecylbenzo)-bis(2,3-pyrido) porphyrazines had increased electron responsibility.

### 2.3. Cancer cell study

The uptake of DMS quaternized zinc bis(1,4-didecylbenzo)-bis(2,3-pyrido) porphyrazines was done in IU-002 cells. IU-002 cells were incubated at 37°C. After incubation for 3 hours, cellular quaternized zinc bis(1,4-didecylbenzo)-bis(2,3-pyrido) porphyrazines was observed with a fluorescence microscope.

A fluorescent substance was noted when the uptake of DMS quaternized zinc bis(1,4-didecylbenzo)-bis(2,3-pyrido) porphyrazines in IU-002 cells was carried out.

Cell rupture can be detected. Intact cells selectively concentrated fluorescence. After exposure to halogen light for 10 minutes showed damage and loss of fluorescence although fluorescence in cells occurred in perinuclear area (see Figure 4).

Concequently, the light exposed DMS quaternized zinc bis(1,4-didecylbenzo)-bis(2,3-pyrido) porphyrazines in cells produces cell disruption that can be detected as a decrease as fluorescence.

### 2.4. Conclusions

Zinc bis(1,4-didecylbenzo)-bis(2,3-pyrido) porphyrazines were synthesized from an equimolar mixture of 3,6-didecylphthalonitrile and 2,3-pyridine carbonitrile in the presence of basic catalyst.

Zinc bis(1,4-didecylbenzo)-bis(2,3-pyrido) porphyrazines having two pyridine and two alkyl-substituted benzene rings reacted with DMS, DES, and MCAA as quaternizing agents.

When MCAA and DMS were employed as quaternizing agents, zinc bis(1,4-didecylbenzo)-bis(2,3-pyrido) porphyrazines were changed to their quaternized derivatives. However, when DES was employed, we showed that sulfonation but not quaternization was achieved.

Electrochmeical characterization of zinc bis(1,4-didecylbenzo)-bis(2,3-pyrido) porphyrazine and its quaternized derivatives were estimated by CV technique. The shapes of CVs clearly showed that quaternized zinc bis(1,4-didecylbenzo)-bis(2,3-pyrido) porphyrazines had increased electron responsibility.

The uptake of DMS-quaternized zinc bis(1,4-didecylbenzo)-bis(2,3-pyrido) porphyrazines was done in IU-002 cells.

The light-exposed DMS quaternized zinc bis(1,4-didecylbenzo)-bis(2,3-pyrido) porphyrazines in cells produces cell disruption that can be detected as a decrease in fluorescence.

## 3. EXPERIMENTAL

### 3.1. Equipment

IR spectra were recorded on a Shimadzu FT-IR 8100A spectrometer using potassium bromide (KBr) pellets. UV-Vis spectra were measured on a Shimadzu UV-2400PC spectrometer; each sample was prepared at 5.0 × 10_−5_ mol dm^−3^ in pyridine, toluene, and water. Fluorescence spectra were recorded in toluene, pyridine, and water using either a Hitachi F-4500 fluorescence spectrometer or a Jasco (Nihon Bunko) FP-6600 spectrofluorometer. ^1^H-NMR spectra were measured at 400 MHz on a Bruker Avance 400S in benzene-d_6_ (C_6_H_6_-d_6_) or chloroform-d (CHCl_3_-d) using tetramethylsilane (TMS) as an internal standard. Elemental analyses were carried out using a Perkin-Elmer 2400CHN instrument. Samples for elemental analysis were purified by repeated sublimation; the instrument was calibrated with copper phthalocyanine. CVs were recorded on a BAS CV-50 W voltammetric analyzer at room temperature in1 × 10^−3^ mol dm^−3^ acetonitrile solution containing a 0.01 mol dm^−3^ tetrabutylammonium perchlorate (TBAP). CVs were recorded by scanning the potential at a rate of 50 mV s^−1^. The working and counter electrodes were platinum wires and the reference electrode was a silver/silver chloride- (Ag/AgCl) saturated sodium chloride electrode. The area of the working electrode was 2.0 × 10^2^ cm^2^.

### 3.2. Materials

TLC was performed using Merck 60 F_254_ **silica gel on aluminium sheets. Merck Silica gel 60, particle size 0.063–0.200 nm 7734 grade was used in chromatographic separations.

Reagents were purchased from Sigma-Aldrich Chemicals (Miss, USA) and were used as received without further purification.

3,6-Didecylphthalonitrile was synthesized from thiophene via 2,5-Didecylthiophene and 2,5-Didecylthiophene-1,1-dioxide, in accordance with our previous reports [9–12]. ^1^H NMR (*δ* 400 MHz, CHCl_3_-d/ ppm) 0.88 (t, 6H), 1.26 (m, 32H), 2.85 (t, 4H), 7.46 (s, 2H); IR(*ν* KBr/cm^−1^) 2960 (*ν*_C–H_), 2240 (*ν*_C≡N_), 1560 (*ν*_C=C_), 1460 (*ν*_C–C_), 1410 (*ν*_C–C_), 1230 (*δ*_C–H_), 730 (*δ*_C–H_); Anal Calcd. for C_28_H_4_N_2_: C. 82.69: H. 10.85: N.6.86. Found: C.82.26: H. 10.84: N. 6.84.

2,3-Pyridine dicarbonitrile was synthesized from pyridine-2,3-dicarboxylic acid. Pyridine-2,3-dicarboxylic acid (15 g, 0.09 mol) in 200 cm^3^ of ethanol was refluxed in 7.5 cm^3^ of concentrated sulfonic acid for 48 hours. After the solvent was removed and neutralized with 3 M NaOH solution, the organic layer was extracted with three times of 75 cm^3^ of diethyl ether. The extract was dried on calcium chloride and filtered and the solvent evaporated. The residue was distilled to afford pyridine 2,3-dicarboxy ester. The ester was dissolved in concentrated aqueous ammonia and the solution was stirred for 48 hours to afford a white-colored precipitate. The precipitate was filtered off and the corresponding diamide was obtained as m.p. 179–181^°^C. Thionyl chloride (9.6 g, 0.08 mol) was added dropwise to the diamide (6.6 g, 0.04 mmol) in 56 g of DMF at −10^°^C. This mixture was allowed to warm to room temperature and was stirred for 24 hours. The solvent was removed by evaporation and water was added to make the thionyl chloride ineffective and then filtered. The product was purified by chromatography over silica gel with benzene-petroleum ether 1 : 1 as eluent, giving a colorless solid (1.97 g, 17%), m.p. 265^°^C.

^1^H NMR (*δ* 400 MHz, CHCl_3_-d/ppm) 7.26 (s, 1H), 7.75 (s, 1H), 9.09 (s, 1H); IR(*ν* KBr/cm^−1^) 3090 (*ν*_C–H_), 2240 (*ν*_C–N_), 1600 (*ν*_C–C_), 1550 (*ν*_C–C_), 1470 (*ν*_C–C_), 1220 (*δ*_C–H_), 750 (*δ*_C–H_); Anal Calcd. for C_7_H_3_N_3_: C. 65.11: H. 2.34: N.32.55. Found: C.65.12: H. 2.34: N. 32.56.

### 3.3. Zinc bis(1,4-didecylbenzo)-bis(2,3-pyrido) porphyrazine

3,6-Didecylphthalonitrile (0.12 g, 0.29 mmol) and 2,3-dicyanopyridine (0.04 g, 0.29 mmol) (see Figure 1) were dissolved in pentanol (7 cm^3^) and zinc chloride (0.05 g) was added; the ensuing mixture was heated for 4 hours in the presence of DBU as catalyst. After cooling, the reaction mixture was dissolved in toluene (50 cm^3^) and filtered; the solvent was removed by evaporation. The product was purified by TLC (eluent : toluene) yielding a blue solid (0.13 g; yield 80%). ^1^H NMR (*δ* 400 MHz, C_6_H_6_-d_6_/ppm) 0.9 (m, 12H, CH_3_), 1.61–2.61 (m, 64H, CH_2_), 4.18–4.36 (m, 8H, *α*-CH_2_), 7.45 (m, 4H, arom), 8.26 (m, 6H, Py); IR(*ν* KBr/cm^−1^) 2960 (*ν*_C–H_), 1600 (*ν*_C–C_), 1500 (*ν*_C–C_), 1420 (*ν*_C–C_), 1200 (*δ*_C–H_), 1100 (*δ*_C–H_), 750 (*δ*_C–H_); UV-Vis [*λ*_max_ toluene/nm (log *ε*_max_)] 665 (5.494); Anal Calcd. for C_70_H_94_N_10_Zn: C. 73.68: H. 8.30: N.12.28. Found: C.73.67: H. 8.30: N. 12.28.

### 3.4. Quaternization of zinc bis(1,4-didecylbenzo)- bis(2,3-pyrido) porphyrazine

**Figure 2:**
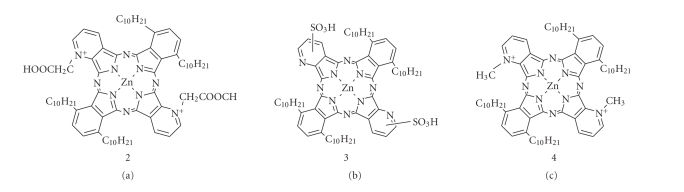
Typical structures of quaternized zinc bis(1,4-didecylbenzo)-bis(2,3-pyrido) porphyrazines.

**Figure 3:**
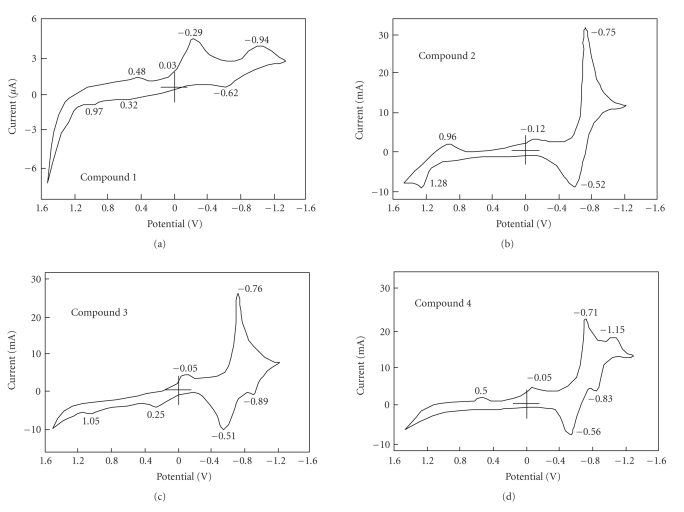
CVs of zinc bis(1,4-didecylbenzo)-bis(2,3-pyrido) porphyrazine.

**Figure 4:**
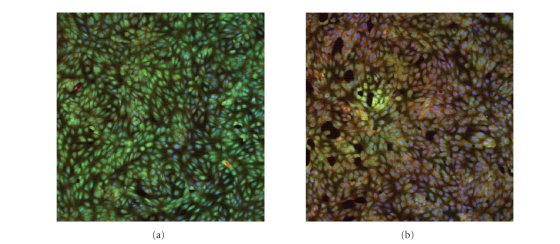
Fluorescence image of IU-002 cells. (a) Control and (b) Incubated with DMS quaternized zinc bis(1,4-didecylbenzo)-bis(2,3-pyrido)porphyrazine and irradiated with halogen light for 10 minutes.

Zinc bis(1,4-didecylbenzo)-bis(2,3-pyrido) porphyrazine (0.17 g, 0.15 mmol) reacted with MCAA (0.57 g, 6 mmol), DES (0.1 g, 0.6 mmol) and DMS (0.2 g, 1.5 mmol), respectively, in *N,N*-dimetylformamide (DMF) 140^°^C for 2 hours (see Figures 2 and 3). The reaction mixture was dissolved in acetone (20 cm^3^), cooled to room temperature, and the resulting solution was filtered. The solvent was removed and the product was purified by TLC (eluent : THF-toluene, 8 : 2); the product was recovered from the TLC plate via dissolution in pyridine followed by filtration and solvent removal. MCAA:Yield 25%, ^1^H NMR (*δ* 400 MHz, C_6_H_6_-d_6_/ ppm) 0.87(m, 12H, CH_3_), 1.13–1.70(m, 56H, *γ*-CH_2_), 1.82–2.61(m, 8H, *β*-CH_2_), 4.11–4.38(m, 4H, *α*-CH_2_), 6.20(s, 2H, CH_2_), 7.14–7.27(m, 4H, Arom), 8.73–.16(m, 6H, Py): IR(*ν* KBr/cm^−1^) 3480(*ν*_O–H_), 3050, 2970(*ν*_C–H_), 1740(*ν*_C=O_), 1600, 1500, 1400(*ν*_C=C_), 1210, 1100, 940, 790, 690(*δ*_C–H_); DES yield 21%, (*δ* 400 MHz, C_6_H_6_-d_6_/ppm) 0.86(m, 12H, CH_3_), 1.02–1.63(m, 56H, *γ*-CH_2_), 1.88–2.61(m, 8H, *β*-CH_2_), 4.26–4.50(m, 4H, *α*-CH_2_), 7.37(m, 4H, Arom), 8.22(m, 4H, Py): IR(*ν* KBr/cm^−1^) 3480(*ν*_O–H_), 3050, 2960(*ν*_C–H_), 1600, 1460, 1400(*ν*_C=C_), 1350, 1150(*ν*_S=O_), 1250, 920, 770(*δ*_C–H_), 580(*δ*_C−S_); DMS yield 25%, (*δ* 400 MHz, C_6_H_6_-d_6_/ppm) 0.90(m, 12H, CH_3_), 0.95–1.45(m, 56H, *γ*-CH_2_), 1.60–2.41(m, 8H, *β*-CH_2_), 4.05(s, 6H, CH_3_), 4.25–4.42(m, 4H, *α*-CH_2_), 7.45(m, 4H, Arom), 8.02(m, 6H, Py): IR(*ν* KBr/cm^−1^) 3070, 2980(*ν*_C–H_), 1500, 1400(*ν*_C=C_), 1250, 1100, 950, 810, 660(*δ*_C–H_).

### 3.5. Cell culture

IU-002 cells were maintained in MEM medium supplemented 5% fetal calf serum.

Cells seeded into 96-well tissue culture plates and incubated to allow attachment to the plates. The sensitizer was added to the medium at concentration ranging from 0 to 2mgcm^3^ . Cells were incubated for 3 hours. The medium was removed, the cells were washed with phosphate-buffered saline (PBS), and fresh medium was added. Cells were exposed halogen light for 10 minutes. Appearance of cells was observed used a fluorescence microscope.

## Figures and Tables

**Table 1:** UV-Vis and fluorescence spectral data of quaternized zinc bis(1,4-didecylbenzo)-bis(2,3-pyrido) porphyrazine.

Compound	Q-band		Fluorescence	
	*λ*_max_ pyridine/nm	*λ*_max_ water/nm	*F*_max_ pyridine/nm	*F*_max_ water/nm
1	687	—	694	—
	^2^665	—	—	—
2	^1^679, 650	^1^687, 647	692	688
	^1,2^677, ^2^620			
3	^1^693, 658, 628, 597	708, ^1^687, 652	698	691
	^2^673, ^2^645, ^2^605			
4	746, ^1^673, 649, 606	723, ^1^676, 646	683	688
	^2^738, ^1,2^668, ^2^641, ^2^600			

^1^Main peak. ^2^In toluene.

**Table 2:** Potentials of quaternized zinc bis(1,4-didecylbenzo)-bis(2,3-pyrido) porphyrazine in DMF with tetrabutylammonium perchlorate.Potentials of reversible wave are midpoint potential of anodic to cathodic peaks for each couple.

Potential (V versus Ag/AgCl)							
Compound	Reduction (*E*_pc_)				Oxidation (*E*_pa_)		
1	–0.94*	–0.62*	–0.29*	–0.03*	0.32*	0.48*	0.97*
ΔE**							
2	–0.75*	–0.52*	–0.12*		0.96*	1.28*	
ΔE**							
3	–0.83	–0.51*	–0.05*		0.25*	1.05*	
ΔE**	0.14						
4	–1.15*	–0.77	–0.14*	–0.05*	0.50*		
ΔE**		0.11					

* Irreversible peak. **The anodic peak to cathodic peak separation for reversible couple.
